# Why do women walk away from maternal health services in Southwest Ethiopia? A qualitative study of caregivers' and clients' perspectives

**DOI:** 10.1186/s12905-023-02207-4

**Published:** 2023-02-24

**Authors:** Sena Belina Kitila, Garumma Tolu Feyissa, Muluemebet Abera Wordofa

**Affiliations:** 1 School of Nursing, Faculty of Health Sciences, Institute of Health Science, Jimma University, Jimma, Ethiopia; 2 Department of Health, Behavior and Society, Ethiopian Evidence Based Health Care Centre, Institute of Health, Jimma University, Jimma, Ethiopia; 3 Population and Family Health Department, Faculty of Public Health, Institute of Health Science, Jimma University, Jimma, Ethiopia

**Keywords:** Maternal health services, Caregivers, Clients, Jimma zone, Ethiopia

## Abstract

**Background:**

Continuum of care for Maternal Health Care is continuity of care through pregnancy, childbirth, and after birth as a key strategy in reaching mothers and babies at a crucial time. Despite the widespread drop out from the continuum of care, there is limited understanding of perspectives of providers and clients about factors leading to drop out from care among women in Ethiopia.

**Objective:**

The aim of this study is to explore the underlying reasons for which women walk away from maternal health services in Ethiopia care providers' and clients' perspectives.

**Methods:**

The population for the study were comprised of all purposefully selected district health department deputy heads, MCH coordinators, primary health center unit directors, midwives and nurses in charge of maternity department and who have been rendering maternal health services and chosen women among those attending the MCH clinic for maternal health services in order to identify reasons for dropout from the perspective of the service users based on the established criteria. The final sample size was determined based on the level of information saturation and a total of 20 in-depth interviewees were conducted. The unstructured key informant interview (KII) guide was used to collect data to gain an in-depth understanding of the context in which continuum of care for maternal health care takes place and existing barriers.

**Result:**

The main themes were identified and compared across all the transcripts to determine similarities and variations in the views of respondents. The major reasons for which women walk away from maternal health services were categorized under three main themes: healthcare system related reasons, community level barriers and individual level barriers. Interpretive analysis was conducted, and elucidations of the results follow the respective themes and verbatim that capture dominant views were considered wherever appropriate to substantiate the findings.

**Conclusion and recommendations:**

Women were walk away from maternal health services because of health system, community level and individual level factors. Hence, implementing initiatives to improve both providers and clients side barriers are essential. Furthermore, we recommend more large-scale studies to digging out more context specific barriers.

**Supplementary Information:**

The online version contains supplementary material available at 10.1186/s12905-023-02207-4.

## Introduction

Continuum of care (CoC) is initially applied in the 1970s and CoC for Maternal Health Care (MHC) is continuity of care through pregnancy, childbirth, and after birth as a key strategy in reaching mothers and babies at a crucial time [1–3].

The COC has recently received attention in maternal, newborn and child health care to ensure that each woman and child receives timely and appropriate care during pregnancy, childbirth and postnatal periods [4, 5].

Regardless of the implementation of a range of maternal health interventions, maternal mortality remains high in many low- and-middle-income countries (LMICs) [6] and dropout from CoC for MHC which is a basic package and critical for women and their infants’ survival and wellbeing is widespread in low-resource settings, with postnatal care visits the most affected [7].

In Africa, each year millions of women die of pregnancy related causes, million babies are stillborn, low birth weight, others live with neonatal complications and many of these problems occur at home, unseen and uncounted in official statistics [8] while key to achieving the Sustainable Development Goal (SDG) target of reducing the global maternal mortality ratio to 70 per 100,000 live births by 2030 [9]

The World Health Organization (WHO) considers Antenatal care (ANC) as a platform for healthcare functions, including health promotion, screening and diagnosis, and disease prevention [10, 11]. Also, mounting evidence, indicates the importance of making the recommended number of ANC visits in increasing use of facility delivery and postnatal care, and the positive correlations among the number of ANC; use of Skilled Birth Attendants (SBA) and postnatal care (PNC) [12–15].

However, there is disparity between the annual number of first ANC visits and deliveries held at a health facility, and the annual number of new ANC visits and the annual number of health facility deliveries is approximately 3:1 or even 4:1 [16].

On the other hand, women who had ANC follow-up, by default, would have access to health facilities for facility delivery and PNC. However, there is a huge disparity across the care pathway in LMICs including Ethiopia [17].

Ethiopia has established a number of strategies and programs to enhance and improve the use of facilities for health care services including MHC [18]. Averting maternal, newborn and child mortality and morbidity is a top priority and a lot has been done both at community and facility level: like capacity building, health facilities expansion, health system governance, human resources, birth preparedness, culture practices, health extension works, social networking, pregnant women forum, maternity waiting homes (MWH) and ambulances to ensure access to skilled providers for the last three decades [12, 15, 19, 20].

Despite all these efforts, the CoC for MHC remains shallow. Research evidence indicates that more than three fifth of pregnant women receive at least one ANC follow-up, yet only one in fifth give birth in health facility [21], According to the EDHS 2019, 47.5% of women gave birth at health care facilities but only 34% had PNC within the first two days, particularly in Oromia where the study was conducted, 41% attended health care facilities for birth and 26% attended PNC within the first two day, but maternal mortality is still high compared to global targets, creating a paradox situation [22]. While the root causes of these are not clearly known, these might be either from the barriers faced by clients, or factors resulting from the perceptions and understanding of the services by the clients or provider-related factors that might affect the quality of care and client satisfaction. Hence, the exploration of the perspectives of both clients and providers qualitatively is crucial in understanding factors leading these dropouts. Evidence on perspectives of providers and clients about factors leading to drop out from care among women in Ethiopia is limited.

Hence, the aim of this study was to explore the underlying reasons for which women drop out from MHC after starting health care utilization.

## Method and materials

### Study setting

This study was conducted at health facilities and offices in the Jimma zone from November to December 2021. Jimma zone is one of the 21 zones of Oromia Regional State, which is situated at 350 kms from the capital, Addis Ababa,in southwest of Ethiopia. The zone constitutes a total of 21 rural districts and two town administrations. According to the Jimma Zonal annual report of the Zonal Health Desk, the zone has a total population of 3,599,836 in the 21 woredas, 562 Kebeles. The zone has, one tertiary hospital, three general hospitals, five primary hospital, 122 Health centers, 512 health posts [23].

### Study design and population of the study

This exploratory qualitative study was aimed to explore factors affecting the continuum of care for MHC from the perspective of both clients and providers.

The population for the study were comprised of all purposefully selected district health department deputy heads, maternal and child health coordinators, primary health center unit directors, midwives and nurses in charge of maternity department and who have been rendering maternal health services for at least one year who were specifically chosen by the investigators based on the established criteria, which includes: their position, proximity to the activities, work experience and consented to be interviewed as key informants.

In order to identify reasons for dropout from CoC for MHC from the perspective of the service users (women), we also included purposefully chosen women among those attending the MCH clinic for maternal health services(ANC, delivery and PNC) during the study period based on their experience of past maternal health services utilization.

Criterion-based purposive sampling technique was used and a total of 20 in-depth interviewees (12 HCPs and 8 women) were conducted. The final sample size was determined based on the level of information saturation (data collection was concluded when we found no new emerging themes) and all the emerging themes were supported through subsequent data collection (Table 1).

**Table 1:** Summary of selected individuals for interview, data sources and methods, November to December 2021, Jimma, Ethiopia

Method	Description	Sample
IDI with woreda health department people	Qualitative interviews with woreda health department people deputy head and MCH coordinator based on their nearness to maternal health services	02 woredas and two individuals per woreda
IDI with health care providers	Qualitative interviews with MCH health care providers	08 Health facilities and one care provider per woreda
IDI with care takers	Qualitative interviews women attending the MCH clinic during the study period	08 Health facilities and one care takers per woreda

### Data collection

We identified individuals who met at least one of the established criteria prior to data collection. The unstructured key informant interview (KII) guide covers information about maternal health services, women’s autonomy, health care-seeking behaviors, the health care environment, socioeconomic barriers, common socio-cultural practices during pregnancy, childbirth and after birth in the community, perceptions towards maternal health care, acceptance of health care providers, their real experience and recommendations that they think it affects CoC for MHC was used for both participants to collect data to gain an in-depth understanding of the context in which continuum of care for maternal health care takes place and barriers to existing health services. The data were collected through face-to-face from an insider perspective using the third person questions to enable respondents to talk about issues without personal attribution. A convenient location (their office and duty station for health care, and maternity waiting area for mothers) was selected by participants and interviewer. The lead investigator/the PhD student and research assistants conducted interviews lasted 30 min on average.

### Data analysis

The field note, interview note, and tape-recorded interviews were transcribed to local language and were later translated into English by a research assistant and lead investigator. Our data was primarily comprised of interview transcripts and field notes.

The fully transcribed audiotaped and field notes of the interview were read and re-read. The transcripts were then given their own color coding, the identified codes were compared across all transcripts to determine similarities and differences. Similar codes were combined and rearranged into higher-order categories. Data and codes were then reviewed in order to determine similarities and differences in respondents' perspectives and themes and subthemes. Accordingly, different main themes and subthemes were found, named, and reported (Table 2). Interpretive analysis was conducted, and elucidations of the results follow the respective themes and verbatim that capture dominant views were considered wherever appropriate to substantiate the findings.

**Table 2:** Summary of characteristics of participants November to December 2021, Jimma, Ethiopia

Characteristics of participants		Number
Age in year	Ranges from 24 to 37	
Sex	Female	10
	Male	10
Marital status	Single	2
	Married	18
Educational level	Diploma	3
	BSc	10
	Other (No formal education, formal education and certificate)	7
Profession	Public officer	3
	Midwives	5
	Nurses	4
Work experience(in years for health care providers)	Ranges from 3 to 12 years	
Occupation of the women	Housewife	5
	Other(gov’t and self-employee)	3
Total	20	

### Data quality maintenance

We recruited and trained data collectors who are familiar with the local culture, who are fluent speakers of the local languages and who have adequate experience in qualitative research method. As the quantitative portion of this study was a prospective follow-up study that increased our familiarity with the setting where the qualitative study was done, we also made an attempt to strengthen the credibility of the findings by getting a rich picture of the context. The role of lead investigator was preparing the proposal, data collection, analysis and interpretation of the data. Similarly, a thick and rich description of the research processes, data collection, analysis and context of the study were reported to maintain the transferability and reflexivity of the study findings.

### Ethical considerations

Ethical clearance was sought and obtained from Jimma University; Institutional review board (Ref.No IHRPGD/433/2019). Participation was entirely voluntary based, and participants were informed that at any time during the interview, they could drop out the study. Participants received an explanation about the purpose of the study, invited to participate, given the opportunity to ask questions for things that may have been unclear and were asked to provide oral consent. De-identification and confidentiality were ensured by using numbers and fictitious names to describe and identify participants.

## Results

We conducted the qualitative study to find out the underlying reasons why do women drop out from maternal health care after starting maternal health care utilization from the care providers and clients prospective among the criterion-based purposefully selected participants. A total of 20 participants took part in the study and their characteristics summarized as follow (Table 2).

Based on the interviews result, we recognized that maternal health services continuation was limited by various motives. However, in this particular paper the identified factors were characterized under three main themes and twelve subthemes. The three main themes that were considered as fundamental reasons underlying why the women dropout across the continuum of care for maternal health care were healthcare system, community and Individual level barriers (Table 3). These themes were analyzed, interpreted and presented; verbatim quotes that represent the broader views of the participants were used to substantiate findings under each category.

**Table 3:** Categories of themes and subthemes, November to December 2020, Jimma, Ethiopia

Theme	Subthemes
Barriers in the healthcare system	Shortfall in supplies and infrastructure (drugs, electric… and water)
	Missed use of the term responsibility and accountability
	High staff turnover and human resource shortage
	Poor linkage and referral system
	Current situation (COVID_19)
Community level barriers	Socio cultural /norm
	Less attention to forum
	Weak community level structure
Individual barriers (women and healthcare providers)	Knowledge and sense of fatigue as GA advanced
	Late booking for ANC
	Loss of motivation
	Socioeconomic

### Barrier 1: health system related factors

The key reasons highlighted by health workers as barriers in the healthcare system agonizing the continuation for maternal health care were: shortage of supplies and infrastructures (drugs, laboratory service, electric power, water supply and functional maternal waiting home), miss use of the term responsibility and accountability, shortages of human resource at facility, care providers turnover, poor communication (poor linkage and referral system) among woreda, health centers and health posts, and miss interpretation of the current situation (COVID_19).

#### Barrier 1a: shortfall in supplies and infrastructures

Supplies and infrastructures are amongst the dares identified as barricades to the continuation of maternal health care in the study areas. High costs of visits to health facilities are associated with transportation issues and difficult road conditions. Transportation costs and the state of the roads were also cited as obstacles to maternal health service utilization. Participants also mentioned a number of facility limitations, such as the malfunction or complete absence of utilities like the water supply, medical equipment’s, electricity and maternity waiting area. These constraints in the facility were among the reasons why service providers did not offer the required level of services, which would have had direct (unnecessary referrals) and indirect (providers’ commitment to offering care) effects on continuity of maternal health care use and one of the care providers claimed this by saying:

Lack of road access, distance and landscape of the area are the known and evident challenges. I [care provider] know that mothers are coming to the health facilities fleeting all these challenges. However, we [care providers] lack supplies to provide them[mothers] with quality services that encourage them [Mothers] to return for more services. For example, currently we [care provider] lack drugs, blood pressure cuff, weight scale, laboratory services, a functional maternity waiting home, electricity and water supply... we [care providers] fetching water from a river using a donkey and or human power, which costs us 20 birrs for a jar containing 20 liters.

[Health care provider at Defkela Health center]

Also, another interviewee from the service users’ side elaborated barriers related to infrastructure saying:

[...] as a resident of Boneya kebele, I have experience to share. A mother was started laboring midnight and we want to take her [the laboring mother] to health center by traditional ambulances. However, she [the laboring mother] gave birth on the way and developed heavy bleeding, she passed away on the way to the health center. Now there is ambulance service. However, the ambulances were unable to reach our kebele during the summer season due to the road condition.

[Caretaker at Boneya Health center]

#### Barrier 1b: responsibility and accountability

Hypothetically knowing the responsibility and accountability allows care providers to strive to perform their daily duties that they are supposed to do and establish a culture of accountability among care providers, feeling comfortable enough to approach a co-worker or manager for help. However, one of the interviewed care providers designated the actuality of erroneous concept of responsibility and accountability in their catchment saying:

[…]If you lie, you will have a better chance of getting an education or a promotion... When you speak the truth and report the truth about what you have done, you risk being demoted or insulted …because of all these reasons we have chosen to lie. Also, if you go up from Primary Health Care Unit [PHCU] to woreda, zone … the common phrase is that the caregivers are not performing their duties. This saying is currently demotivating caregivers and preventing them from performing their duties as expected. Even they [care providers] are aware of this saying and confirmed it in local language by saying "Oogeessi hojii keessa hin jiruun hojii keessaa nubasee."

[Health care provider at Korjo, Health center]

Other care provider also said:

In my opinion, there is no system that holds healthcare providers accountable based on evidence; rather, every thinning is based on devotees. Care providers in a PHCU are divided into three groups: supporters of the PHCU director, opponents of the director, and those in the middle. This categorization results in a lack of team spirit in the facility, less engagement in community mobilization, friendly service, advancing or counseling clients to return to the facility, and a compromise of service quality, which pushes the community away from service utilization rather than pulling.

[Health care provider at Odo Hidhata Health center]

#### Barrier 1c: shortage of care providers and staff turnover

Obviously, the shortage of manpower and turnover results in disrupts in service operations, loss of experienced and trained staffs, fatigue and negligence, unnecessary referrals, failure to respond to women’s preference, dissatisfaction with the work climate, imbalanced composition of new and experienced staff that leads to difficulty in arranging work schedules, increased workloads and provision of lower quality services, all of which contribute to discontinuation of the services utilization. Despite the fact that the ANC period is regarded as an ideal time to discuss the continuum of maternal health care, listen to the women's concerns and provide them the proper information on when and where to go as well as what they need to do. On the other hand, shortage of human resources may prevent health professionals from giving patients enough health information. According to one respondent:

[…] we are working for longer hours (24 hours) due to the shortage of manpower... let me tell you about the scenario of this month …this month there is only one midwife working in three units: ANC, labour and delivery and family planning. Surely, I do not believe he is providing these services, screening, effective reassure and counseling as expected to be given due to duty overload and a lack of time, and these substandard services, less information, less engagement in community mobilization may result in discontinuity of maternal health service utilization.

[Health care provider at Defkela Health center]

On the other hand, the interviewed mothers described as the mothers seeks much information and advice from care givers, and one of the pregnant mothers reflect her feelings saying:

[…] our women are not well educated and need follow-up and advice from care givers …in short uneducated women are like a metal, and she [ the woman] addressed this with the proverb “wallaalan akkuma sibilaati yoo ta’anii , ta’anii moorodan malee” which means that what you sharpened last year will not stay sharp forever, , so our mother need continue follow-up and awareness creation like metal sharpening process.

[Pregnant mother,Odo Hidhata health center]

#### Barrier 1d: poor communication (poor linkage and referral system) among woreda, health centers and health posts

One of the barriers to low utilization of maternal health services and drop out was the lack of strong linkage and communication among woreda, health centers, and health posts. This is explained by the following quote from a health care provider:

“In theory, health extension workers (HEWs) are expected to work in accordance with their (HEWs) packages. However, they are forced to manage other non-health sector activities...spending a significant portion of their time on non-health sector activities. For example, they are currently collecting government taxis and working on HEWs-related activities only twice a week, which is our bigger problem”

[Health care provider at Korjo Health center]

Other care provider also said:

“…Practically the mothers are expected to receive ANC2 and ANC3 at health post level, but we lack strong communication with HEWs. Even in this case, it is difficult to know who completed their ANC and who did not, and I know this from the report”

[Health care provider at Alle Health center]

Other care provider also mentioned:

“As to me [care provider], our health systems communication is seasonal and active when only top administrative level communication is available, when the regional health bureau awakens, the zone, woreda, and PHCUs are too otherwise …”

[Health care provider at Asendabo Health center]

#### Barrier 1e: miss interpretation of the current situation (COVID_19)

COVID _19 has been identified as one of the barriers to the utilization of maternal health care in a variety of ways. The effect of COVID 19 was explained in this study by the interruption of pregnant women mobilization by HEWs through home-to-home visits, the interruption of pregnant women forums, the modified transportation regulation during this period that limited /reduced the number of passengers per vehicle by half and increased the cost of transportation, the health information given to society about COVID 19, and the effect of COVID 19 on pregnant women.

The quote from one respondent was:

[...] Yes, we discussed these issues [problems related to maternal health service withdrawal] in detail with woreda health administration, kebele leaders, and kebele managers last year. However, due to COVID_19, there is no mobilization of pregnant women for services through home-to-home, and the transportation cost, which was five birrs previously, has now increased to 25 birrs, as these mothers are from low-income families , they do not come for these services.

[Dedo woreda health office].

Also, another pregnant mother elucidated this by the quote:

[...]You [caretakers] are teaching us [Mothers] about COVID 19 and its effect on pregnant women... Personally, I am afraid to visit a health facility because I don't know who is infected, and I will only do so if I have no other choice.

[Mother from, Alle Health center]

### Barrier 2: community level factors

Sociocultural factors, trust in care providers, less attention to forums, and a weak community level structure (such as the women's developmental army, the male developmental army, and the Gare) were identified as community level barriers to the continuation of maternal health care utilization.

#### Barrier 2a: sociocultural factors

Because of a variety of factors such as time of labor onset, not knowing their gestational age, geographical and societal norms, most women who give birth at home are assisted by their elder untrained women in the community. This is demonstrated by the following quote from one of the women participants:

Most of us [the mothers] had no idea about our [the mothers'] gestational age, and what you [care providers] told us [the mothers] was far from our [the mothers'] time of labor delivery. Furthermore, it is culturally unallowable for us [the mothers] to leave the home after the onset of labor, even to go to the health facility for labor delivery, due to the fear of what we [the community] locally refer to as "Michii," which complicate the labor delivery process. As a result, we [the mothers] prefer to give birth at home.

[Pregnant mother from Defkela health center]

Other care providers elucidated by the quote:

[...] In my experience, home delivery is perceived as normal, whereas facility delivery is appropriate for the sick or those who have complications. Similarly, the majority of the mothers did not know their [the pregnant mothers'] gestational age and EDD, which we [care providers] tell them [the pregnant mothers], but this is usually varies from their [the pregnant mothers'] actual time of delivery, and sudden onset of labor could also be one of the barriers for them [the pregnant mothers'] not delivering at facility.

[Health care provider at Asendabo Health center]

Other care providers also said:

During delivery at night, the caregivers usually tie the hand torch to the head or hold it in the mouth. This method [tying the hand torch to the head or holding it in the mouth] directly projected the light into their [women's] birth canal, this imposes discomfort and causing mothers to avoid coming to the health facility for delivery.

[Omo Nada, Woreda health office]

The client's trust in the care provider's advice and treatment is an essential component of the care provider–client relationship. Untrust in providers may exacerbate disparities and lead to the discontinuation of maternal health services.

This is explained by the quote from one of the interviewees:

The victimed person is the right person to detect his/her own problem and to care for him/herself , which they [mothers] locally called “Of maramaruuf abbuma wayyaa …our communities culturally do not allow women to leave the home until they are 49 days postpartum, and … we call it locally “Gaaddidduu”

[Health care provider from Alle Health center]

#### Barrier 2b: forum pregnant women forum

The interviewees acknowledged the lack of awareness creation program in the health care facilities and community level including pregnant women forum and a regular meeting of the Women Development Army.

This is explained by the quote from one of the participants:

[…] It is healthier if the community receives more information about complications during pregnancy, as well as during and after delivery... they [mothers] will understand more about these complications [danger signs], and most of the time our community does not pay attention to problems after delivery.

[Mother from, Asendabo Health center]

### Barrier 3: individual (mother and HCPs) level factors

Women's knowledge of their gestational age (GA), fatigue as GA progressed, misinterpretation of concepts, late booking for ANC, loss of motivation, and socioeconomic status were amongst the accentuated individual (maternal and health care providers) barriers for continuation of maternal health care.

#### Barrier 3a: feeling tiredness

The women also shared their own experiences saying:

As gestational age advances, I [the women] become tired to walk a long distance on foot to reach health facilities and we [the women] the access to the health facility is what you know and see. You [care provider] also know what will happen as the gestational age advances.

[Pregnant mother from Metoso health center]

#### Another pregnant mother elucidated this by the quote:

[…] Personally, I [pregnant mother] have difficulty walking and don't have the same power that I had during the ANC1 compared to when my gestational age advanced and I got closer to term, and this is my main difficulty in continuing to use my services.

[Pregnant mother from Defkela health center]

#### Barrier 3b: late booking for ANC and Miss interpretation of the concepts

One of the reasons for not completing the MHC was the late booking for ANC due to various contributing factors. In this study, critical concerns include a lack of awareness about ANC services, a lack of complete MHC service packages at health facilities, and how care providers report the findings of their assessments and investigations.

One of the health centers MCH coordinator explained saying.

[…]yes, this[MCH] is our [care givers] concern too, the mothers are not coming back for ANC4 after ANC1, if you look at our reports, we are reporting more than 100 ANC1 per months but, delivery and ANC4 were much less; this could be related to the approach we are using [ANC 2 and ANC 3 are given at health post level by HEWs] may be among the reasons why they[women] drop out the follow up based on our[caregivers] observations.

[Health care provider from Metoso health center]

Another pregnant mother elucidated this by the quote:

[...] After the investigations, they [caretakers] told us [the mothers] the results of the examination, saying there is no problem, in local language "Rakkoon hin jiru"... Since a woman is told there is no problem, most of them [women] believe there is no problem and why they[women] bother to get back to health facility without have a problem. For me this is among the barriers why women drop out their follow up.

[Mother from, Gudeta Bula Health center]

#### Barrier 3c: loss of motivation even we have no linkage with HEWs

The lack of infrastructure, like nature of the road, distance, location of the health center, distance of health post from health centers and topography of the woreda, availability of transportation, and availability of service packages at facility were known barriers for underutilization and continuity of maternal health services, and now one of the emerging barriers was dishonesty, which we [care providers] refer to in locally language: Sobanii barnoota argachuu, dhugaa gabasanii harrabsamuu… Kanaaf soba filanne and the majority of us [the caregivers] have been following the 59 principles, which means start working 11:00 a.m. and leaving at 3:00 p.m.

[Health care provider from Korjo Health center]

#### Barrier 3d: socioeconomic

Despite the fact that maternal health care services are provided for free, one of the identified barriers to the continuation of maternal health care service uptake in this study is the cost of the services. It was evident from the study that cost, both health and non-health expenses, are significant obstacles to obtaining maternal care services in the study area. In this regard, it was noted that only small number of well-to-do individuals in the community have access to health facilities. Frequent drug stock-outs in the health facilities require that women have to purchase prescribed medication, which is sometimes unaffordable for families with limited resources.

One of the key informants said.

[…]also, after my assignment here, I taught that the indirect cost, the cost mothers pay for transportation, is much higher than the direct cost, and we call it the user-fee exemption policy for maternal and child health services, which has been less successful in making access to MHC equitable across economic categories.

[Health care provider from Asendabo Health center]

## Discussion

Diverse studies showed that almost nine out of ten women dropped out from the pathway of the continuum of care for MHC [24–26]. The current study was aimed to contribute to the body of knowledge on continuum of care for maternal health care through exploring the perspectives of both providers and clients on the reasons for which women drop along the path for maternal health care.

We found three thematic categories of reasons for the dropout of women along the path for maternal health care were categories. These includes (a) health system, (b) community level and (c) individual level factors.

The findings of the current study are partially in agreement with the findings of the study conducted in Assosa Zone, Benishangul Gumuz and Northwest Ethiopia which found socio economic, knowledge of maternal health service, poor roads. poor health facility readiness, lack of ambulance, cultural and traditional beliefs, providers being male, women’s education level, urban residence, media exposure, perceived time to reach health facilities and unprofessional behaviors as the major barriers for the maternal health service uptake [27, 28].

The identified factors in this study also concur with other studies in sub-Saharan Africa and has provided further insights into the barriers for continuation of maternal health care like the health system level factors (erroneous concept of responsibility and accountability, high staff turnover, poor communication), sociocultural (*“Michii” and “Gaaddidduu”),* Individual level factors (miss interpretation of concepts (COVID_19,“Rakkoon hin jiru”), knowledge about gestational age, late booking for ANC, dishonest *…kanaaf soba filanne,* the 59 principles) and the indirect cost for maternal and child health services which are often not covered in national level intervention strategies and programmatic actions.

This study also showed that high staff turnover which may be connected with the lack of electricity and other infrastructure. Poor or absence of infrastructures affects recruitment and retention of qualified health personnel in the communities which in turn indirectly affect access to significant maternal health services particularly in rural area where health care systems are challenged by fresh care providers which may be leads to dropout from attending healthcare services the care and women seeking care from unexperienced providers [29].

The Ethiopian government has also subsidized MCH services to make them affordable and available. Despite this initiative, this study showed that the indirect cost for maternal health care is by far higher than the direct cost that limited women’s access to these services and continuation.

Given that healthcare facilities are less equipped with the necessary skilled manpower and equipment, clients’ experience at the facilities might not be positive. In addition, the low incentive system for the healthcare workers in rural remote areas will not motivate them to satisfy the needs of mothers coming for delivery. This is especially critical as the healthcare workers are also expected to attend large number of deliveries, especially during the night and weekends. The poor referral system also creates further burden on women, especially in the context of the poor transport infrastructure. The transportation conditions usually worsen during rainy season and at nights time as transport providers hike their tariffs beyond what most members of the community can afford, which is an unexpected indirect cost that may also contribute to the drop out from the continuum of care. For instance, if the ambulance is unavailable the pregnant women are conveyed by inappropriate transport like commercial motorbikes, this may exacerbate their already deprived health conditions. Delays in getting pregnant women to the referral facility sometimes led to fatalities and near-misses, this condition [fatalities and near-misses] may be used as a confirmatory for the sociocultural views like “Michii” in the community. Hence, in addition to posing discomforts and additional economic burden, the health care seeking process, especially for labor and delivery within the context of such poor infrastructure is burdensome. In addition, the entire system both in the healthcare and beyond is not addressing the cultural, physical and psychological needs of the rural women.

In general, this study found that the continuum of care is influenced by complex factors both within and outside the health sector, which have a negative impact on the family's and the country's health and health care costs.

However, there are some limitations to this study. The findings cannot be generalized because they are based on qualitative data. There may be social acceptance bias, in which participants respond in the way that they are more likely to be accepted. Additionally, this study solely examines scenarios in primary care institutions; there was no facility observation; and the study's scope does not include system limitations in referral and teaching hospitals.

## Conclusion and recommendations

The reasons for which women drop from maternal health services are related to health system, community level and individual level factors. This needs the preparation of care and robust health systems strengthening. In addition, addressing infrastructure related barriers is crucial.

Within the healthcare system, implementing initiatives to improve both client-side barriers and providers side barriers such as staff shortages, motivation, supplies, logistics and health facility capacity are essential. Creating habitable conditions and enabling environments for health care providers, implementation of an evidence-based, culturally responsive health care system is critical to reducing this dropout. Furthermore, we recommend more large-scale studies to dig out more context specific barriers.

## Supplementary Information

**Additional file 1. **COREQ (COnsolidated criteria for REportingQualitative research) Checklist.

## Data Availability

The datasets generated and analyzed during the current study are not publicly available but are available from the corresponding author on reasonable request.

## Abbreviations

ANC: Antenatal care
IRB: Institutional Review Board
MCH: Maternal Child health Care
LMICs: Low and Middle-Income Countries
PNC: Postnatal care
SBA: Skill birth attendance
SDGs: Sustainable Development Goals
WHO: World Health Organization
